# Keep Your Mouth Shut
*"Keep Your Mouth Shut. A Popular Treatise on Mouth-Breathing: Its Causes, Effects, and Treatment." By Fred A. A. Smith, M.D., C.M.Glas., M.R.C.S.Eng., Hon. Surgeon Cheitenham Eye, Ear, and Throat Infirmary. (London: Baillière, Tindall, and Cox.)


**Published:** 1893-01-14

**Authors:** 


### New Books and New Editions.

### "KEEP YOUR MOUTH SHUT."*

Under this frank and imperative title Dr. Smith treats of the proper method of breathing, and of the dangers that arise from using the mouth for a function which belongs to the nose. The chief of these is that the air which would be warmed to the temperature of the blood if it reached the lungs by way of the nasal passages arrives there too cold when inhaled by the mouth, and tends to set up inflammation. The milder *sequelæ*, such as snoring in sleep, need not be too severely handled, as this is not fatal, save to the comfort of a bedfellow. Dr. Smith adds to the numerous anxieties which beset a mother's life by warning her that a fall may so displace the interior arrangements of a child's nose as to set up mouth-breathing at once, and to lead, indirectly and remotely, to nasal polypus. In case of such an accident surgical treatment will be required to restore the equilibrium, but it is a fortunate circumstance that the nose is such an unimportant part of a child's face that the inevitable bumps fall mostly on cheeks and forehead. Besides the subject of his title-page Dr. Smith deals with infant ophthalmia and the folly of employing untrained midwives, which he condemns with much earnestness and no little reiteration. He assures us that the latter is intentional, and is meant to impress upon mothers the dangers which they and their children encounter at the hands of the ignorant Gamp; but such mothers are not very likely to meet with this book. As a matter of fact the essay could well stand condensation, and would in all probability attain more practical usefulness if published in pamphlet form.

* "Keep Your Mouth Shut. A Popular Treatise on Mouth-Breathing: Its Causes, Effects, and Treatment." By Fred A. A. Smith, M.D., C.M.Glas., M.R.C.S.Eng., Hon. Surgeon Cheltenham Eye, Ear, and Throat Infirmary. (London: Baillière, Tindall, and Cox.)

